# GamePlan4Care, a Web-Based Adaptation of the Resources for Enhancing Alzheimer’s Caregiver Health II Intervention for Family Caregivers of Persons Living With Dementia: Formative, Qualitative Usability Testing Study

**DOI:** 10.2196/60143

**Published:** 2025-07-11

**Authors:** Jinmyoung Cho, Thomas Birchfield, Jennifer L Thorud, Marcia G Ory, Alan B Stevens

**Affiliations:** 1 Center for Applied Health Research, Baylor Scott & White Research Institute, 2401 S 31st St, Temple, TX, 76508, United States, 1 2542159795; 2 Saint Louis University School of Medicine, St. Louis, MO, United States; 3 Texas A&M School of Public Health, College Station, TX, United States

**Keywords:** dementia caregivers, user interface/user experience (UI/UX), technology, web-based caregiving program, user experience, training, online resource, peer support, Alzheimer disease, dementia, caregiving, caregiver, carer, informal care, family care, spousal care, usability, clinical trial, aging, elderly, geriatrics, gerontology, mhealth, mobile health, digital health, online platforms, web health, online support, neurodegeneration, brain degeneration, memory loss

## Abstract

**Background:**

The negative consequences of caregiving can be mitigated by providing caregivers with support programs that increase their dementia care skills and provide emotional and tangible support. Web-based technology can increase the availability of evidence-based caregiver interventions. GamePlan4Care (GP4C) is a web-based adaptation of the Resources for Enhancing Alzheimer’s Caregiver Health II (REACH II) intervention, redesigned and reformatted for web-based delivery.

**Objective:**

The goal of GP4C is to create a web-based family caregiver support platform that facilitates self-directed exposure to evidence-based skills training and support for caregivers of persons living with dementia. This multidimensional approach of using technology enhanced with live support has the potential for improved scalability and sustainability. In preparation for a randomized clinical trial of the new intervention, the GP4C platform underwent user interface/user experience (UI/UX) testing with caregivers as part of an iterative design process.

**Methods:**

UI/UX testing of caregivers’ reactions to technical and content-related aspects of the platform was conducted with 31 caregivers recruited through partnerships with community-based organizations in central Texas. Usability testing consisted of performing system tasks, answering open-ended questions on the tasks, and providing feedback on their experience with the platform. Two researchers used an inductive thematic approach to data analysis using transcripts of individual audio and screen-recorded sessions with each participant. The analysis consisted of 3 phases: data familiarization, coding, and theme formulation.

**Results:**

In total, 18 participants tested technical-related aspects of the GP4C platform, and 13 participants tested content-related aspects. The average age of participants was 62 (SD 12.2, range 31‐86). A majority of participants were female (27/31, 87.1%) and White or Caucasian (26/31, 83.1%) while almost one-third were Hispanic (10/31, 32.3%). The thematic analysis revealed 3 themes: supportive resources as a common theme, active engagement for technical aspects of the platform, and a comprehensive approach for content aspects of the platform. Participants also suggested changes in navigation and content.

**Conclusions:**

Findings from the usability testing sessions indicate that the platform provided engaging, useful content that the caregiver would continue to use, resonated with their caregiving experience, helped the caregivers think through their choices and emotions, and could be used to help communicate with the person living with dementia. Caregivers appreciated the personalization based on what they had already completed and the concept of having a Dementia Care Navigator when they needed additional help. Caregivers also provided multiple suggestions on how to improve the system, including changes for easier navigation and inclusiveness. This positive feedback indicates that with a few changes, the platform would be beneficial to meet the needs and provide resources for caregivers of persons living with dementia. The process of involving end users in usability testing during the development stage ensures that the finished tool will better meet users’ expectations and current needs.

## Introduction

### Background

Communities across the United States are challenged to meet the physical, emotional, and social needs of persons living with dementia. Currently, nearly 7 million Americans are living with dementia, and that number is expected to almost double in the next 25 years [1]. While efforts to increase the availability of formal health and social care services are essential, support of family caregivers is paramount if they are expected to continue to provide the vast majority of daily care and support of persons living with dementia. As reported in the National Academies’ report, Families Caring for an Aging America, nearly 70% of caregivers in a nationally representative survey had provided 2 to 10 years of care, and 15% had already provided care for more than 10 years at the time of the survey [2]. In other words, an average person in their fourth decade of life is expected to eventually spend 5.0 years (or 10% of their remaining life) caring for an older adult, a situation known to have negative consequences [2]. This is significant because caregivers are more likely than noncaregivers to experience negative consequences on their well-being such as increased depression, anxiety, burden, social isolation, and family conflict [3-5].

The negative consequences of caregiving, however, can be mitigated by engaging caregivers in programs that increase their dementia care skills while providing emotional and tangible support. A recent systematic review shows that caregivers are more likely to engage in and benefit from multicomponent interventions that assess the caregiver’s challenges as well as their emotional response to providing care [6]. These considerations respect the unique circumstances of each family caregiving situation and lead to a tailored program of education, skills training, and support [7-9]. While caregiver interventions that use a tailored approach to education, skills training, and support exist, services derived from these evidence-based interventions are not routinely provided by health and social support organizations. Two major barriers to the provision of such services include the cost of developing a workforce trained in evidence-informed dementia care strategies and the lack of formal payment systems (eg, commercial insurance and Centers for Medicare and Medicaid Services) [10-12].

Web-based technology provides an alternative approach for moving evidence-based interventions into widely available caregiving education, skills training, and support programs. Web-based caregiver support programs have the potential to increase the availability of caregiver interventions that have previously been delivered through an in-person or telephone format [13,14]. Moreover, web-based technology can be designed to meet the expressed desire of caregivers for an on-demand, self-paced, learning and skills training experience to support their role as a dementia caregiver [15]. Use of web-based technology in the provision of existing evidence-based interventions is supported by research on caregiver interventions designed for web-based or mobile delivery [16-19].

Multiple meta-analyses of caregiver interventions designed for internet delivery have shown promise, but findings have been mixed [13,20-22]. Some reviews found that technology-based interventions were just as effective as face-to-face interventions; however, mixed delivery methods showed greater improvements than web-based, telephone, or DVD-based interventions alone [21]. Leng et al [13] found that personalized internet-based interventions had a greater effect size than nonpersonalized interventions on depressive symptoms and perceived stress. These findings suggest the need for more attention to the engagement of family caregivers in the design and user testing of web-based platforms for dementia caregivers.

To address this need, the research team created a web-based family caregiver support platform, GamePlan4Care (GP4C), by applying technology to an existing evidence-based intervention (ie, Resources for Enhancing Alzheimer’s Caregiver Health [REACH II]). This study explored the usability of GP4C among family caregivers of persons living with dementia.

### GP4C: A Web-Based Adaptation of REACH II

GP4C incorporates a risk-based approach and aligns the self-reported needs of a caregiver with therapeutic content consistent with the REACH II domains of safety, stress, health, emotions, care services, support, and behaviors. Similar to REACH II, the GP4C therapeutic strategies include educational materials, tools (eg, worksheets), skills training exercises, and access to peer support. Importantly, the web-based format of GP4C allows strategies to also be presented in video format, which was not done in REACH II. The therapeutic process is facilitated by a Dementia Care Navigator, a trained interventionist who assists the caregiver in the navigation of the web-based content and personalized goal setting via telephone and email support.

After an initial build of the platform to include the functionality of an initial assessment of the caregiving situation followed by access to the safety and emotions domains of the GP4C content, we conducted caregiver usability testing on these domains to identify additional design needs in preparation for a randomized trial. This manuscript describes the caregivers’ experience in using the initial design of the GP4C platform and key aspects of how they experienced the functionality of the platform regarding the initial assessment and tailored education and skills training on the 2 domains of GP4C: Safety and Emotions.

## Methods

### Process

The GP4C research team worked with a large integrated health care system’s Digital Health Department to develop a task hierarchy cataloging individual tasks to accomplish within the platform and salient design questions appropriate for experimenter prompts. Using phenomenological methods, the research team conducted usability testing concurrently with content enhancement via a method of iterative evaluation using the think-aloud technique [23]. This method characterizes the ease with which a user can complete a task, by what means a user attains mastery of system features, and problems a user encounters while using the system. Such tasks included registering, logging in, answering user assessments, and navigating to education and skill-building content (Textbox 1). Participants tested either the technical-related aspects (user interface and design) or content-related aspects (wording or appropriateness of questions and feedback, satisfaction with education and skill-building content) of the GP4C platform. User testing sessions for each participant were audio and screen recorded. Participants performed system tasks while vocalizing their thoughts, feelings, and satisfaction with the platform. Research staff prompted the participant with a question to elicit specific feedback regarding that feature. Upon completion, participants also provided their overall impression or opinions regarding their experience with the platform. Participants were not given access to a Dementia Care Navigator as part of this user interface/user experience (UI/UX) testing.

Textbox 1. **Summary of tasks that were tested by the participant, a caregiver of a person living with dementia, during GamePlan4Care usability testing.**

**Technical-related aspects**

- Logging into the platform
- Responding to questions regarding the context of caregiving
- Managing messaging feature
- Reviewing automated feedback
- Assessing video content
- Managing goal progress

**Content-related aspects**

- Logging into the platform
- Responding to questions that assessed caregiving risk associated with the domain
- Reviewing two core skill videos
- Reviewing three related exercise videos
- Reviewing materials, including worksheets

### Recruitment

Participants for the usability testing were recruited through referrals from local partnerships with community-based organizations (such as Area Agencies on Aging and Alzheimer’s focused not-for-profit organizations) from October 2019 to February 2020. Program champions at our partner organization recruitment sites identified interested participants and sent referrals through a secure Research Electronic Data Capture (REDCap; Vanderbilt University) website after receiving verbal consent by the interested participant. Upon receipt of a referral, research staff reached the interested participant by telephone to complete eligibility screening. If eligible, interested participants scheduled a usability testing session at a recruitment site. Informed consent was conducted electronically at the time of the usability testing session immediately before the intake assessment and usability testing occurred. Interested participants received information about the study from the program champion prior to verbal consent to be referred and from research staff during the eligibility screen telephone conversation.

### Participants

Eligibility criteria for this study was for caregivers to be 18 years of age or older, reside in our recruitment area, be able to read and speak English, provide care or supervision to a friend or family member for an average of at least 8 hours per week for the last 6 months, and have access to a home computer or tablet with internet access and use it for an average of at least 3 times per week. The care recipient, or person receiving care, must also have a diagnosis of Alzheimer disease or a related dementia (self-reported diagnosis by the caregiver was accepted) and have signs or symptoms of dementia (defined as a score of 2 or greater on the Ascertain Dementia 8, [24]). Criteria that excluded caregivers from participating included being currently enrolled in another evidence-based caregiver education and support intervention.

Program champions referred 113 individuals to the program, of which 102 were distinct, unduplicated referrals. Of these, 61 individuals were screened for eligibility by the research team and 41 were not screened (1 care recipient passed away, 29 were unable to be reached, and 10 were not interested, had time constraints, or were unable to travel for the testing session). Forty individuals were eligible for the intervention and 21 were ineligible. Reasons for being ineligible included the care recipient not having a dementia diagnosis (n=9), participating in another caregiving intervention (n=5), living outside of the recruitment area (n=2), not having access to or using a home computer (n=2), and not providing care for at least 8 hours per week (n=3). Reasons for not enrolling include time constraints (n=2) and other reasons (n=7). A total of 31 caregiver participants completed the sessions, 18 were recruited to complete user testing of the technical-related aspects of the platform and then an additional 13 were recruited for testing of the content-related aspects.

Tables1  2 summarize the demographic characteristics and caregiving experience of participants. A majority of participants were female (27/31, 87.1%) and White or Caucasian (26/31, 83.9%). The average age of participants was 62 years old (range 31‐86). Nearly a third of participants were Hispanic (10/31, 32.3%). Caregivers were providing care to a variety of family relations with a majority being parents (-in-law) or spouses (26/31 participants, 83.9%).

**Table 1.** Demographic characteristics of GP4C^a^ usability testing participants (n=31, caregivers of persons living with dementia) recruited from October 2019-February 2020.

Participant characteristics	Values	
Age, mean (SD)	62.0 (12.2)	
Sex, n (%)		
Female	27 (87.1)	
Male	4 (12.9)	
Race, n (%)		
White/Caucasian	26 (83.9)	
Black or African American	3 (9.7)	
Other or more than 1 race	2 (6.5)	
Hispanic ethnicity	10 (32.3)	
Marital status, n (%)		
Married or living as married	20 (64.5)	
Widowed	2 (6.5)	
Divorced or separated or never married	9 (29.1)	
Employment, n (%)		
Full-time	8 (25.8)	
Part-time	2 (6.5)	
Homemaker	2 (6.5)	
Retired	13 (41.9)	
Other	6 (19.3)	
Relationship to care recipients, n (%)		
Parents (-in-law)	15 (48.4)	
Spouses	11 (35.5)	
Brother, grandfather, or friend	5 (16.1)	

a GP4C: GamePlan4Care.

**Table 2.** Caregiving experience of GP4C^a^ usability testing participants (n=31, caregivers of persons living with dementia) recruited from October 2019–February 2020.

Caregiving characteristics	Values, n (%)	
CR^b^ diagnosis		
Alzheimer disease	13 (41.9)	
Dementia	15 (48.4)	
Vascular dementia or TIA^c^	4 (12.9)	
Parkinson’s disease	2 (6.5)	
Lewy-body disease/Fronto-temporal disease	5 (16.1)	
MCI^d^	2 (6.5)	
Distance to CR		
Living with CR	4 (12.9)	
Less than 15 min	22 (71.0)	
Within 15‐30 min	1 (3.2)	
Within 1 hour	4 (12.9)	
Indirect care hours per week		
Up to 8 hours	1 (3.2)	
9‐19 hours	2 (6.5)	
20‐39 hours	3 (9.7)	
40+ hours	23 (74.2)	
Direct care hours per week		
Up to 8 hours	1 (3.2)	
9‐19 hours	5 (16.1)	
20-39 hours	10 (32.3)	
40+ hours	15 (48.4)	
Caregiving duration		
6 months - 2 years	9 (29.0)	
2‐5 years	13 (41.9)	
5+ years	9 (29.0)	

a GP4C: GamePlan4Care.

b CR: care recipient.

c TIA: transient ischemic attacks.

d MCI: mild cognitive impairment.

Participants reported that the majority of care recipients were diagnosed with either Alzheimer disease (13/31, 41.9%) or dementia (15/31, 48.4%). Over 80% of caregivers were living with the care recipient (4/31, 12.9%) or living nearby (less than 15 minutes; 22/31, 71.0%). Most participants also reported providing over 20 hours of either indirect (26/31 participants, 83.9%) or direct (25/31 participants, 80.6%) care weekly for at least 2 years (22/31 participants, 71.0%).

### Data Collection

Usability testing sessions were conducted by 2 members of our research team (one male senior project manager, author TB, and one female research project coordinator). Both were masters-trained researchers with psychology and public health backgrounds with training in counseling and research ethics. Sessions lasted approximately 2 hours with each participant. Only the participant and the member of the research team were in the room for the usability testing. Research staff read each task individually to the participant with prompts about each task. Throughout the testing session, research staff prompted the participant with generic prompts to explain what was happening during the testing and the participant’s thoughts. At the end of the testing session, research staff asked participants questions about their overall impression of the platform, what could be improved, and the positive and negative aspects of the platform. Research staff had blank paper available during the usability testing sessions for any notes that could be used in future analysis of the participant’s performance while completing the tasks, including an overall sense of the participant’s computer literacy.

### Analysis

Analysis of the data was performed using an inductive thematic analysis approach [25] with 31 transcripts using no statistical software. The research team focused on participants’ progress on the tasks and their valuable feedback on their experience as they completed the tasks. Participants were observed, vocalized what they were thinking and feeling, and then provided final feedback after completion of the tasks. All of this information was triangulated to determine the users’ experience with the platform [26].

Computerized transcripts were reviewed initially for quality assurance by research staff. Two researchers completed the thematic analysis. Both researchers independently reviewed the transcripts and revisited the recorded audio of participants completing the tasks. Initial ideas were documented and discussed during the initial phase of data familiarization. The coding phase of the data was then initiated. Key terms (words and short or long phrases) were identified to illustrate the concepts of the caregivers’ experience and feedback found in the transcripts and were saved in an excel spreadsheet. Key terms were discussed over multiple meetings. Once agreement was reached on the key terms, they were brought to the full team for further discussion. These key terms represented underlying ideas from the data that the research team considered pertinent to the aim of the study. The third stage consisted of formulating themes by grouping together key terms that reflected similar overarching ideas. Common themes from technical and content-related aspects and a major theme specifying each aspect emerged. In addition, participant-suggested themes also emerged. Under each major theme, subthemes related to the main theme, but separate and distinct enough to be differentiated, were also revealed. Major themes and subthemes applied to the data were defined and labeled. Finally, examples were chosen from the transcripts to illustrate theme elements. IBM SPSS software was used for descriptive statistics of the participants [27].

### Ethical Considerations

This study was approved by the institutional review board at the participating institution (Baylor Scott & White Research Institute, IRB Number 018‐622) and participants gave informed consent. Informed consent was given electronically before data collection and usability testing, and participants were given a paper copy to follow along with during the electronic consent process. Participants chose to receive either an electronic or mailed copy of the signed consent form. All data presented are deidentified. Participants received US $100 gift cards after completion of the usability testing.

## Results

### Overview

**Figure 1.**
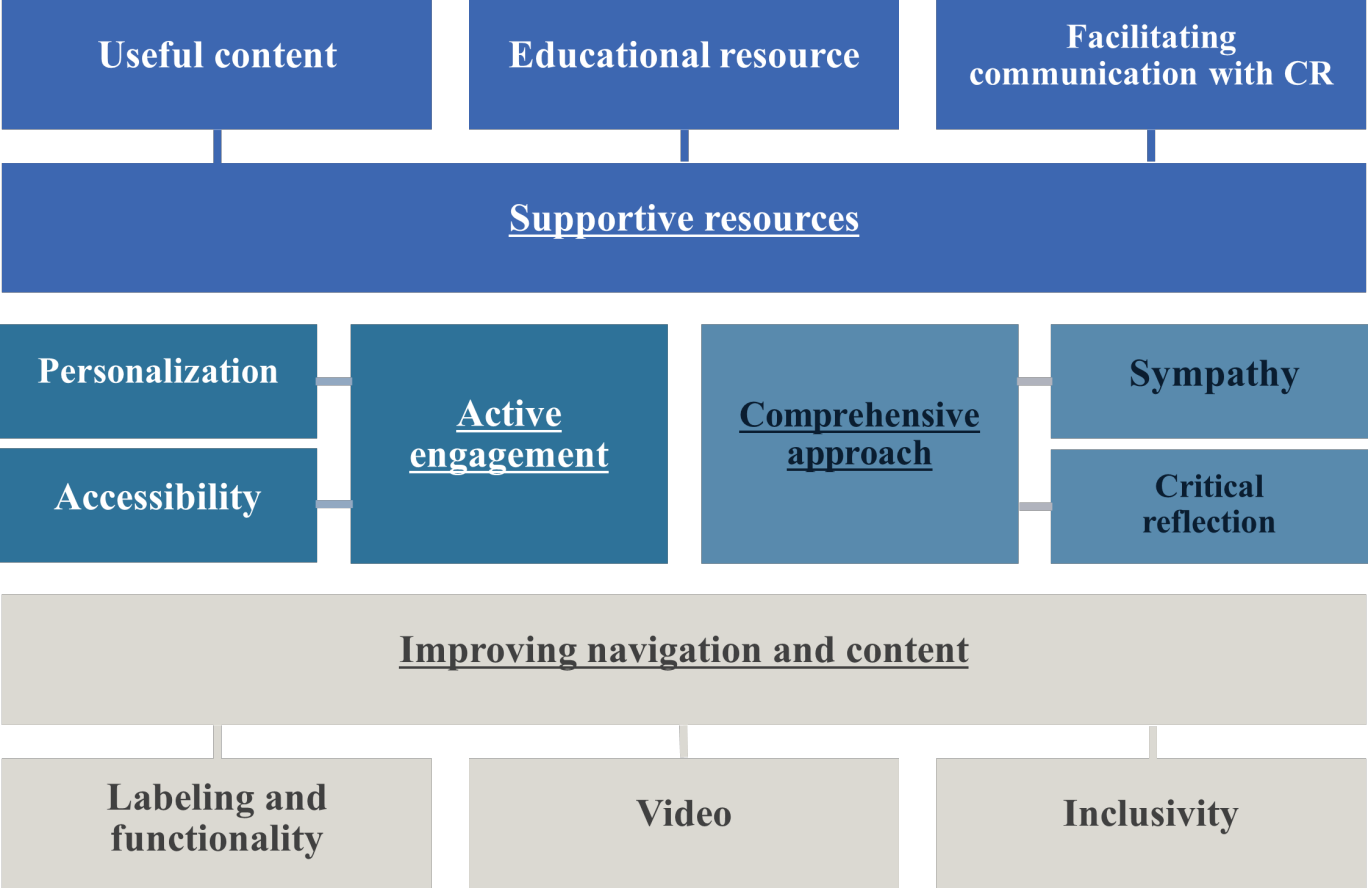
Summary of major themes and subthemes of the GP4C usability testing data from caregivers of persons living with dementia recruited from October 2019–February 2020. Note: Main themes are underlined. GP4C: GamePlan4Care, CR: care recipient.

Overall, many participants seemed satisfied with the quality of the content and flow of the platform. One participant said, “Oh, it is very clear and understandable…” Another participant commented that “it [videos] weren’t exhausting or boring.” Another participant felt that the videos “…are very clear, very simple, they are not overwhelming, they are not over my head, so it is good.” Several other participants also commented that the videos were “very soothing.” A participant also mentioned that they thought the videos would be used by caregivers to refer back to when brainstorming different strategies to use with the care recipient. An overview of the themes and subthemes is presented in Figure 1.

### Common Theme: Supportive Resources

In addition to the general positive responses, all the participants agreed that the platform provided very supportive resources for their caregiving responsibilities. Caregivers described the content as useful and educational, suggesting that the platform would be beneficial to facilitate their caregiving tasks and communication with their care recipients.

#### Useful Content

Participants felt that the content was appropriate. When they were asked to review the safety topic in particular, some caregivers seemed to agree that the safety tips on driving are an important strategy that they should address with their care recipients. For example, one caregiver said, *“*It is very clear that there is going to be some strategies and…hints to help the caregiver.” Another participant noted, “I am liking what it says. This is a reminder of what I need to be looking for.*”* Similarly, another participant explained, “It is not too long, yet it is very informative.” Another participant echoed a similar perspective by saying, “I think this is very good as far as different topics and stuff.*”* Another participant commented that the content was “conversational” and that they liked the “vocabulary” used throughout the platform. One participant expressed that they felt the videos were useful, they said, “…reading something is one thing, but to have the video show, it just really keeps your mind, you know, wanting to continue.”

#### Educational Resource

Several participants commented that the program would help them persist with caregiver education. For example, one participant said, “It is not something I would just watch and put it away. I could have these printed out in my little book.” Another participant stated, “I like how they are almost making it [the program] like a continuing education.” Similarly, another participant said, “It’s not too long, it gives information, it makes me want to come back and see what else is there.”

#### Facilitating Communication With Care Recipients

As many participants recognized the materials as helpful and educational, a participant commented that the worksheets could help facilitate difficult conversations with the care recipient and help them decide on changes together.

*So, to discuss those worksheets…that’s another good reason for worksheets… I'm saying I feel like I'm always the one to having to say no you can't, no you can't, and so the person receiving care, this is a way… to say we're going to go through this and we're going to help you identify what things are safe for you, what you feel comfortable with and what you may not. So, it’s a platform that opens up the communication with that individual to try to keep them safe as it relates to driving.*

### Technical Theme: Active Engagement

Participants noted that they appreciated the active engagement required for dementia caregivers. One participant said that they liked “That you are actually trying to do something to help the caregivers out there rather than just saying oh you got a local support group down the street.” A caregiver also mentioned that she appreciated she could access the information on her own time. She said, “…it is giving you support where there is nothing expected of you, you know you don’t have to come back and do 20 visits, it is all online when you have time.” Another participant felt similarly and said, “I like the thought of tutorials that I can go through and work on myself at my pace and all that.” The feature that the information was gathered and organized in one central location was appreciated. For example,

*I see this [is] very unique and different than I’ve accessed so far, and I’ve been doing a whole lot of research…I’ve been online for weeks and week and weeks now, so this is definitely something that I think will be used a lot…*

#### Personalization

Participants liked the level of personalization. For example, after entering information about the care recipient, caregivers would go on to receive questions that included that relationship (eg, “your mother”). One participant said, “the most positive are definitely the suggestions that strictly apply to me from what they heard from my answers.” Similarly, another participant said, “It is nice to know that they have recognized my answers.”

#### Accessibility

Participants also seemed to appreciate that they could connect with a professional (ie, dementia care navigator) for additional help, especially in times of crisis. A participant said,

*It would be good to have [a dementia care navigator], as a caregiver you get flustered with everything and bogged down with everything and you cannot think correctly sometimes because you are overwhelmed with trying to figure out how to fix the situation.*

Another participant said,

*I think that that’s important, but there are some times where you just feel alone because there is nobody out there who gets what you are going through…and you have a specialist out there who is willing to listen without judgement…*

One participant also stressed the importance of a care specialist (or other professional) providing thoughtful and specific feedback when reviewing information that had been completed on the platform. They said,

*I would want to know that somebody was reviewing and understanding what my situation is. I wouldn’t want somebody to be, you know, just looking at it and doing a cursory review.*

### Content Theme: Comprehensive Approach

The themes from content interviews or sessions can be divided into 2 subthemes: sympathy and critical reflection. Caregivers expressed that the features of the platform resonate with their experience.

#### Sympathy

A participant appreciated that the topic they visited (ie, caregiver guilt) was featured in the videos, which resonated with their experience. The participant explained,

*I’m very happy to hear that guilt is something that they are definitely touching on…guilt is a big part, and as a caregiver, you can find yourself going down that road…very quickly.*

Another participant also appreciated that addressing caregiver guilt was included in the platform. The participant said,

*I like that [it’s included] because…sometimes you feel guilty and it’s just a little bit, but sometimes you feel guilty and it’s extreme.*

Another participant agreed and said,

*When I feel guilty I feel like God how can I be tired or I will think to myself…you know she took care of you and lot of thoughts…but maybe [I] felt ashamed because I felt that and then of course I felt sorry.*

Similarly, another participant commented that

*Another thing…that needs to be said here is that you’re going to have negative feelings and you’re going to have the negative responses, and you have to learn to forgive yourself. I think because in the sense telling me I shouldn’t have the negative thoughts increases my guilt.*

#### Critical Reflection

Caregivers also felt that the videos did a nice job of helping caregivers think critically about their choices. A participant said, “…it makes you think where can they drive, what do I cut out first.” Another participant commented that the video made them think differently about their emotions. The participant said,

*sometimes I think ‘mom, are you just playing with me?’…they are not, you just got to be patient and then the second part of it about the guilt in that, that really makes you think and makes you analyze yourself.*

Several caregivers also noted that they appreciated that the videos reminded caregivers that they could not change the care recipient. A participant said,

So, I also very much appreciate the way that they are subtly reminding the us as caregivers that the person with dementia cannot change and that is a very big part of dealing as a caregiver for with someone with dementia and Alzheimer’s is that they don't know how they are and have to be…to remind yourself this is not who the person was. This is who they are now, and they cannot change it. So, I like seeing that in here.

### Participant Suggested Themes: Improving Navigation and Content

As a main goal of the tasks, participants provided feedback on how to improve the platform features and contents. Three areas of improvement were identified: labeling and functionality, video, and inclusivity.

#### Labeling and Functionality

Clearer labeling or using different colors on the platform was suggested by several participants. For example, incorporating a “box that says next” rather than just an arrow. Another participant suggested “the button area and the fonts” could be bigger. Another participant suggested making completed sections “a different color than the rest” and several participants mentioned that they were color blind and had a difficult time seeing text show up. The participant said, “…I’m color blind, so text itself, I can’t tell what that looks like until I go to the box.” However, other participants commented that they liked the colors that were used. One participant said,

*Oh, I like all the blue and gold and stuff like that. I think they are very soothing to the eye. The whites good because you need to make everything else pop up on the screen better. I mean, I think this is good because at least the dark blue highlights all the stuff over here.*

#### Video

One of the few criticisms of the videos was given by a participant who felt that the videos were “a little slow in some parts.” However, another participant disagreed and felt that at times the narrators in the video were “speaking a little fast.” Another participant suggested that the video reflections have more parameters. For example, they suggested that the video narrator say, “tell me what you’re thinking in two sentences” rather than “what are you thinking?” One participant felt that the video narrator should introduce themselves each time and provide a brief overview of the topic. The participant felt this would be particularly helpful when caregivers were skipping around the site. They said, “…maybe you need to … tell what you’re going to talk about.”

#### Inclusivity

Participants addressed the importance of making sure the materials were inclusive for a wide range of individuals. For example, a caregiver also noted that cost efficiency is important when creating any materials that caregivers are expected to print. The caregiver said,

*I have to control cost in the household, because I’m looking at hiring people to come in, so if I can print this, even though this looks pretty, if it would print in black only without me having to do anything, then that would be great because this is extra ink and cost.*

Another participant mentioned concerns about how a caregiver might access materials, particularly if “some of the [computer] software is really, really old.” The caregiver went on to explain that if it was too complicated, caregivers would likely not download the worksheets and that “technical support” may be useful for some caregivers. One participant also mentioned the materials were needed for individuals who didn’t speak English. The participant said, “…within the United States there are many Spanish-speaking individuals, so at some point certainly should be translated…”

## Discussion

### Principal Findings

This study presents participants’ experiences from user testing of GP4C, a web-based adaptation of the REACH II caregiver intervention. Similar to other usability studies [28,29], both content- and technology-related aspects of our platform were explored. We focused on the way participants experienced and reacted to the GP4C platform while completing their tasks. Results showed that GP4C was appreciated by participants because it offered a unique resource for those who provide care to a person living with dementia. In general, participants recognized the platform’s comprehensive approach reflecting their experience as caregivers. Active engagement, including personalization for their own needs and accessibility to resources such as the Dementia Care Navigator intervention staff, was emphasized as the best features of the platform. After completing tasks with the platform, participants provided valuable suggestions to improve navigation and platform content. Thus, the overall positive responses from participants were promising and encouraged the research team to move forward in finalizing the platform.

### Implications of Platform Revision Findings

The qualitative findings of the study were used by the research team to revise the platform before testing in a randomized clinical trial. The following design changes were implemented in the final iteration of the GP4C platform.

#### Navigation and basic layout

Based on user feedback, the research team worked with the development team to change the navigation and basic layout. For example, the platform included explicit buttons of “Next” and “Back,” which were resized and recolored to increase visibility, callouts (eg, “new message”) were added to explicitly capture the caregiver’s attention, and clues (eg, images, spinners, and prompts) were added to make workflow and progression smoother.

#### Assessment items and response choices

Not surprising, the participants preferred a conversational tone in assessment items and real-life reflective responses. For instance, one assessment question was changed from “How severe are the dementia symptoms?” to “How would you describe his or her thinking and memory changes?” and one of the choices to the question, “Does he or she drive?” was changed from “Never” to “Never, or not anymore.” This feedback from participants was considered in the development of the risk assessments being tested and for the additional 5 content domains that were created after UI/UX testing.

#### Tools to orient users to the site and its use

Some participants, especially older caregivers, were frustrated with the newly developed interface because they were not familiar with it. To assure simplicity and logical progression of the interface, the platform added more tools to assist caregivers (eg, welcome video presented upon first visit to user dashboard, opening and closing scenes to remind users how to start and complete goals, and demonstrations of live use of worksheets). Each of the 7 content domains included in the final version of GP4C shared a similar format in presentation of information, support in goal setting, and functionality.

### Comparison of Findings to Existing Platforms Designed for Caregivers

Usability testing of other web-based caregiver support interventions found findings similar to our study. Feedback from two websites, one web-based training and support program for dementia caregivers [30] and one informational website for people with dementia and their carers [31], indicated that changes needed to be made to the labeling and functionality of “next” buttons [30,31], indicating a need to ensure easy readability and navigation. A systematic review of web-based interventions for dementia caregivers found similar findings regarding the importance of the use of text for labeling, font size, and color choice for buttons [32]. Similar to our platform personalizing content for users, the program by Teles et al [30] allowed users to self-personalize the platform by completing a user profile, which was found to be appraised positively. A usability testing of a web-based intervention for informal caregivers [33] and an acceptability and feasibility pilot study of a self-guided, web-based intervention for dementia caregivers [17] found, similar to our results, caregivers appreciated the use of video content and thought the videos were engaging and helped communicate key takeaways in a timely manner. The systematic review by Ottaviani et al [32] also found that the use of videos and practical examples with real care situations was valuable to users. One area of recommendation from the usability testing of a web-based program for caregivers of persons living with Alzheimer disease that our platform included in its design and implementation was the ability for users to interact with professionals [34]. Accessing a professional was a planned component of the program by Cristancho-Lacroix et al [34], but was not implemented due to a lack of resources. A feasibility study of a web-based intervention for dementia caregivers included motivational coaching sessions incorporated into their platform [35]. Feedback from participants indicated that they greatly appreciated the availability of a coach through multiple means (video call, phone call, and email) and thought the personal feedback provided by the coaches increased their active engagement with the intervention [35]. Another qualitative study of a web-based intervention for caregivers of older adults with dementia and multiple chronic conditions found that participants recommended the availability of a person to help answer questions and direct the participant to resources that met their specific needs [36]. These findings support the benefit of the inclusion of a Dementia Care Navigator in our platform. Although web-based interventions are designed to be self-directed, users appreciate the ability to have access to a professional when they need additional support.

### Limitations

This exploratory usability testing is not intended for generalization but rather to provide user feedback to a specific digital health tool. Thus, a limitation of this study may be its lack of representativeness in the sample and generalizability to a broader population. The participants of the study were recruited from one geographic area of the United States. Other caregiver populations in different regions might present different opinions due to their accessibility to local resources and support. Next, the themes identified and presented in this study were identified in the midst of the GP4C platform development. Participants were only allowed to visit portions of the domains (ie, safety and emotions) rather than the full platform. This was because the iterative design resulted in updates being made to the platform as the usability testing was being completed. We acknowledge that participants may express different opinions on the final version of the platform if they were able to visit all of the domains. Moreover, given the limitations of the sample size, demographic variation in participants was not included when analyzing findings. Furthermore, the digital divide may lead to voluntary bias among caregivers from diverse backgrounds. The participants who provided feedback for this study had internet access and were already inclined to use it for web-based information searches. Additional recruitment efforts should be made to minimize this bias. Despite these limitations, this study provides a model for conducting usability testing of web-based caregiver support programs with the end user.

### Conclusions

This study presented the first stage of the development of GP4C, a web-based family caregiver support platform. This study demonstrates the value of having end users participate in the development and early evaluation of digital tools. The UI/UX approach provided feedback on both the technical and content aspects of the web-based platform developed by the research team. Caregiver feedback led to changes in the functionality of the system and guided the research team in the development of additional content to be added to the system. Usability testing and participants’ in-depth and positive feedback showed its functionality and utility as a potential caregiver support program for dementia caregivers. The research team finalized the platform based on these results and is currently conducting a randomized clinical trial to show the effectiveness of the platform.

## Abbreviations:

GP4C: GamePlan4Care
REACH II: Resources for Enhancing Alzheimer’s Caregiver Heath II
REDCap: Research Electronic Data Capture
UI/UX: User interface/user experience
